# Gastrointestinal parasitosis in cattle: Unveiling the landscape across diverse production systems in Bangladesh

**DOI:** 10.1002/vms3.1325

**Published:** 2023-11-27

**Authors:** Md. Abu Sayeed, Lauren Ungar, Yeasin Haider Chowdhury, Md. Saiful Bari, Md. Mizanur Rahman, M. Sawkat Anwer, Md. Ahasanul Hoque

**Affiliations:** ^1^ National Centre for Epidemiology and Population Health The Australian National University Canberra Australia; ^2^ Cummings School of Veterinary Medicine at Tufts University Medford Massachusetts USA; ^3^ Chattogram Veterinary and Animal Sciences University Chattogram Bangladesh

**Keywords:** commercial farm, gastrointestinal, parasitosis, prevalence, resistance

## Abstract

**Background:**

Factors influencing parasitosis in cattle in Bangladesh remain inadequately explored, necessitating a comprehensive investigation for interventions and sustainable livestock farming.

**Objectives:**

We conducted this study to estimate the prevalence and distribution of gastrointestinal parasites, exploring their intricate relationship with farm management practices across a spectrum of small‐, medium‐, and large‐scale commercial farms.

**Methods:**

We conducted this study in the Chattogram district of Bangladesh. We collected a total of 189 freshly voided faecal samples from different farms. We recorded the age, breed, milking status, sex, body condition score, and anthelmintic use history of the sampled animals. We processed the samples using the direct smear method, with the identification of one egg per sample being considered positive.

**Results:**

We estimated the prevalence of gastrointestinal parasite infection in large‐scale (52.1%), medium‐scale (54.5%), and small‐scale farms (70.0%), with statistically significant differences (*p* ≤ 0.05). Both pregnant and lactating cows, as well as indigenous cattle, were more likely to have gastrointestinal parasites (*p* ≤ 0.05). The predominant parasites across farms of all sizes were trematodes (*Paramphistomum* spp. and *Schistosomas* spp.) and protozoa (*Balantidium coli* and *Coccidia* spp.).

**Conclusion:**

Poor farm management practices, such as no pasture management and inadequate deworming regimens, may contribute to the elevated prevalence and infection load observed on small‐scale farms. The increased parasitosis in previously dewormed animals can be attributed to the development of anthelmintic resistance against gastrointestinal parasites. Implementing proper and effective deworming strategies is crucial to preventing gastrointestinal parasitosis and mitigating the risk of anthelmintic resistance.

## Graphical abstract

We examined the pasture management techniques and gastrointestinal parasite prevalence in Chattogram district of Bangladesh. We obtained a total of 189 freshly voided faeces samples and examined using different laboratory evaluation techniques. Small‐scale farms exhibited the highest gastrointestinal parasite prevalence, notably in pregnant and lactating cows as well as indigenous cattle. High prevalence correlated with suboptimal pasture management and deworming practices. Implementing effective deworming protocols is crucial to preventing gastrointestinal parasite infections and combating parasitic resistance.

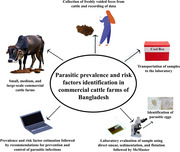

## 1 INTRODUCTION

Bangladesh is a densely populated country, with ∼80% of the rural population rearing cattle or any other livestock (Kamal et al., 2019). The total cattle population of Bangladesh is 23.8 million (DLS, 2017) and 1.5 million in Chattogram (Md. Reajul Huq, District Livestock Officer, Chattogram, personal communication., 2018). Compared to neighbouring countries, Bangladesh has low productivity in both meat and milk production (FAO, 2005). Total milk and meat production was 9.3 and 7.2 million metric tons, respectively; there was a deficiency of 5.6 million metric tons of milk and a surplus of 0.02 million metric tons of meat (DLS, 2017). Cattle are extremely important to household farmers; they are directly linked to the family's income, welfare, and nutritional intake. It is important to maximize production and improve animal health to provide the most output for farmers at all levels.

The cattle rearing system in Bangladesh is mostly a traditional free‐ranging system, but currently a commercial rearing system is gaining popularity. Categorically, a large‐scale commercial dairy farm is one that contains ≥26 cows and where the farmers have considerable experience farming and produce a certain number of dairy cows with uniform average milk production around the whole year (Halder & Barua, 2003). A medium‐sized commercial farm contains 6−25 cows; the number of cows may vary from month to month as well as milk production throughout the whole year. Small‐scale or household farms are those in which people rear less than five cows in an open or semi‐closed shed, providing minimum veterinary care and artificial breeding, and milk production is low at around 1450 kg per cow per year (Hamid & Hossain, 2014). Small‐scale dairy farming is one of the important facets of village economies in this country (Mumba et al., 2011). In Bangladesh, the majority of the total 6 million cattle, ranging from approximately 85% to 95%, are indigenous, while crossbred cattle make up 10% to 15% (Hamid & Hossain, 2014). Notably, among the crossbred cattle, there is a predominance of species like the local‐Holstein Friesian cross. Additionally, the cattle population includes Red Chittagong cattle and various exotic breeds (e.g. Friesian, Jersey, and Sahiwal), observed in different areas of Chattogram (Md. Reajul Huq, District Livestock Officer, Chattogram, personal communication., 2018).

Rates of parasitism throughout Bangladesh are high due to its geographical position with a low‐laying area, heavy rainfall, and humidity (Hossain et al., 2016). Cattle are mostly susceptible to gastrointestinal parasites (GIPs), which have negative effects on the productivity and health of ruminants (Adua et al., 2017). GIPs cause calf mortality (Bilal et al., 2009), enteric diseases (Van Metre et al., 2000), decreased growth (Jittapalapong et al., 2011), lowered productivity (Zvinorova et al., 2016), and reduced milk production (Charlier et al., 2009). These negative effects pose an economic risk to farmers and the country's economy as a whole (Biffa et al., 2006). Infection by GIPs may be ignored or overlooked due to the lack of outward signs (Zafar et al., 2022). The reported prevalence of GIPs in cattle in Bangladesh, ranging from 72.0% to 84.8%, and in India, a neighbouring country, varying from 61.1% to 81.8%, reflects the prevalence among the cattle and buffalo population across different months of the year (Ahmed et al., 2015; Alim et al., 2012; Marskole et al., 2016; Nath et al., 2016). However, the comparison of different types of parasitic infection and their prevalence among Bangladeshi cattle in different production systems has not been previously studied in a structured way.

Recognizing the significance of environmental and individual animal‐level risk factors, encompassing ecology, geography, climate, age, sex, breed, and season, this study aims to bridge existing knowledge gaps by investigating the prevalence, associated risk factors, and distribution of GIPs.  Notably, the role of farm management practices, such as cleaning frequencies and biosecurity measures, in the context of parasitosis has been understudied in Bangladesh (Ahmed et al., 2015; Akanda et al., 2014). Therefore, this study seeks to address this research gap, contributing insights into the prevalence, associated risk factors, and distribution of GIPs in the context of diverse farm production systems.

## 2 MATERIALS AND METHODS

### 2.1 Description of study sites

The areas of Mirarsarai upazilla (sub‐district) and Khulshi thana of Chattogram were selected for this study. While Mirarsarai is characterized by hilly coastal terrain, Khulshi exhibits a semi‐hilly landscape. Chattogram is at an elevation of about 29 m and experiences average annual temperatures, humidity levels, and rainfall ranging from a minimum of 13°C to a maximum of 32°C, 70−85%, and 5.6−727.0 mm, respectively (Sayeed et al., 2017). Both regions host cattle within diverse production systems, including small‐scale or household setups, as well as medium and large commercial production systems.

### 2.2 Selection of study farms

We selected the dairy farm unit within Nahar Agro Industry, Mirarsarai (large scale) (355 cattle), nine medium‐scale farms (160 cattle; minimum: 8; maximum: 24), and 21 small‐scale household farms (64 cattle, minimum: 2; maximum: 5) from Khulshi to investigate the cattle parasitosis during July–August 2018. The Nahar cattle unit was chosen specifically due to its status as a modernly equipped commercial cattle farm. The medium‐scale commercial cattle farms were recruited based on the herd size of at least six cattle per farm and farmers’ willingness to participate in the study. The small‐scale household farms were chosen based on a herd size range of 2–5 cattle per farm and the farmers’ willingness to participate in the study.

### 2.3 Selection of cattle

For this study, we randomly selected 189 cattle, with 94 (N = 355) originating from the large‐scale commercial farm, known as the Nahar cattle unit. Additionally, 55 (N = 160, from 9 farms), and 40 (N = 64, from 21 farms) were selected from medium‐scale farms and small‐scale or household farms, respectively. We omitted those calves that were fed milk or milk replacer from the sampling. Other than those, to ensure that every animal had an equal chance of being chosen, we did not apply any explicit inclusion or exclusion criteria during random sampling. Considering 1% precision and 99% confidence, we computed the sample size based on the prediction of an expected prevalence of 11.9% (Ahammed et al., 2017) using online tools (http://www.openepi.com/Menu/OE_Menu.htm).

### 2.4 Faecal sample collection, preservation, and transportation

We have aseptically collected a total of 189 fecal samples from the rectum or freshly voided faeces of selected animals under different production systems. Approximately 15‐20 grams of faeces were collected, placed in a sterilized bottle with a unique identification number, and transported to the laboratory within 3 hours of collection using an ice‐cool box. Before laboratory analysis, the samples were preserved at 4°C in the laboratory.

### 2.5 Data collection

We have collected epidemiological data from every farm through a questionnaire. We recorded the age, breed, status (milking or dry, pregnant, or non‐pregnant), sex, body condition score, and anthelmintic dosing for each sampled cow. Farm management and biosecurity measures were also recorded. Farmers’ interviews, farm record books, direct physical examinations, and observation of the animals were used to obtain the necessary information. We designated the breed exactly as the breed data recorded from the farms containing crossbreeds, indigenous breeds, Hariana, and Sahiwal. We classified the animal classes according to the dataset, which contained bull, heifer, calf, milch cow, pregnant heifer, pregnant dry cow, and pregnant milch cow. Deworming status was reported as anthelmintics of any type administered in the studied animal (within the previous 4 months from the study day) or not.

### 2.6 Sample processing and lab testing

We examined all samples using the direct smear method for the presence and identification of parasite eggs (Urquhart et al., 1996). In brief, we deposited a few droplets of water and an equal quantity of faeces on a microscope slide. We tilted the slide to enable the lighter eggs to separate from the heavier debris, placed a cover slip over the fluid, and then examined the preparation under a microscope. All negative samples underwent further examination by simple sedimentation for the presence and identification of eggs. In brief, the faecal sample was homogenized in water to achieve uniformity, and the suspension was then strained to remove fine particles and debris. We transferred the filtrate into a conical flask and allowed it to stand for 2 minutes before removing the precipitate and transferring the remaining 15 mL of filtrate into a separate tube. After an additional 2 minutes of sedimentation, the supernatant was discarded. We transferred a small volume of the remaining sediment to a microscope slide using a dropper or pipette, and after adding a coverslip, we examined the sample under the microscope (Urquhart et al., 1996). A third examination by flotation using a sugar and salt mixture either confirmed or negated the negative samples. Faecal samples having at least one egg of any type of parasite or cyst per sample on microscopic examination were considered positive. They were further examined using the modified McMaster Counting technique (Kassai, 1999; Soulsby, 1968) to determine the parasitic concentration and density (egg per gram or oocysts per gram).

### 2.7 Data entry and statistical evaluation

We managed both field and laboratory data using Microsoft Excel 2007, where we debugged the data and ensured fidelity. Subsequently, the data were exported to STATA‐IC‐13 (StataCorp, 4905) for executing statistical analysis.

#### 2.7.1 Descriptive analysis

We categorized the distribution of parasite eggs per gram (EPG) in faeces into different groups within different production systems, and then computed the proportion and frequency numbers. However, we categorized the EPGs previously by percentiles (None = 1; 1–100 = 2; 101–200 = 3; 201–16,000 = 4) as they were not normally distributed even after log transformation. We calculated the proportionate prevalence of gastrointestinal parasites in different production systems (small‐scale or household and medium and large‐scale commercial farms) based on the number of GIP cases according to EPG categories. Finally, we presented an overall gastrointestinal parasite proportionate prevalence under different production systems by summing up the percentage of GIP cases for each EPG category. Simultaneously, we applied the same descriptive statistical approach (frequency, number, and percentage) to outline the status of other categorical variables, including group and parasitic types, level, and types of gastrointestinal parasitic infections under different production systems. Contrarily, we computed summary statistics for continuous variables such as parasite eggs per gram of faeces under different factors, including production type, breed, animal class, sex, and deworming.

The Kruskal–Wallis test was conducted, followed by Dunn's post hoc test, to discern a univariate association with the status of EPGs. The level of significance was determined as *p* ≤ 0.05. The Dunn test estimated the statistical relationship between two categorical variables under each factor.

#### 2.7.2 Risk factor analysis

Although the response variable was quantitative (EPG), the risk factor analysis was done based on a dichotomous outcome. Therefore, if the sample tested positive for parasitic eggs with a minimum number of EPG (>0), the subsequent cattle were recorded as positive, while those not meeting these criteria were identified as negative for GIP infection (yes or no). A *χ* ^2^ test was conducted to assess the difference in GIP infection among categories of individual risk factors.

To identify risk factors, a univariate logistic regression analysis was performed for the prevalence of GIP infections. The exposure variables were farm types, animal class, breed, sex, and deworming (yes or no). Before running univariate regression analysis, the farm types were regrouped as commercial, combining large‐ and medium‐scale farms versus small‐scale farms (household) to ensure an appropriate frequency distribution for each category. The animal class was regrouped as calf versus bull and heifer versus milch cow versus pregnant dry and milch cows for the same reason. The same procedure was followed for breed categories (cross vs. indigenous) as well. The level of statistical significance was set at *p* ≤ 0.05. The outputs of the univariate logistic regression analysis were displayed as odds ratios (OR) and 95% confidence intervals (CI).

## 3 RESULTS

### 3.1 Gastrointestinal parasite egg status in cattle across different production systems in Chattogram

We estimated that the overall proportionate prevalence of GIPs in cattle based on the samples evaluated by McMaster technique (minimum 160,00 eggs per gram of faeces: EPGs) was 52.1% (*n* = 49) in cattle of the large commercial farm (N = 94), 54.5% (*n* = 30) in cattle of the medium‐sized commercial farms (N = 55), and 70.0% (*n* = 28) in cattle of small‐scale or household farms (N = 40). These results significantly varied among production systems (*p* ≤ 0.05).

We estimated that the proportionate prevalence (PP) of GIPs at the category of minimum‐100 EPGs was 6.4% (*n* = 6) in cattle of the large commercial farm, 34.5% (*n* = 19) in cattle of the medium‐sized commercial farm, and 20.0% (*n* = 8) in cattle of small‐scale or household farms, which differed significantly among different production systems (*p* ≤ 0.05). In the category of 101−200 EPGs, the PP was 19.1% (*n* = 18) in cattle of large‐scale commercial farms, 16.4% (*n* = 9) in cattle of medium‐sized commercial farms, and 30.0% (*n* = 12) in cattle of small‐scale or household farms, which significantly varied among production systems (*p* ≤ 0.05). At the category of 201–16,000 EPGs, the estimated PP was 26.6 % (*n* = 25) in cattle of large‐scale commercial farms, 3.6% (*n* = 2) in medium‐scale commercial farms, and 20.0% (*n* = 8) in cattle of small‐scale or household farms, which also significantly varied among production systems (*p* ≤ 0.05).

### 3.2 Association of gastrointestinal parasite EPGs in cattle and selected factors in Chattogram

We estimated that the median of EPGs of GIPs differed significantly by production type, animal classes, and deworming status (*p* ≤ 0.05) (Table 1). Cattle from large‐scale commercial farms exhibited the highest median EPGs (300), followed by cattle from small‐scale or household farms (200) and cattle from medium‐scale farms (100). Among different breeds, Hariana and Indigenous cattle displayed the highest median EPGs (250‐300). Among animal classes, pregnant animals had a higher median of EPGs (300–450) (Table 1). Notably, no significant variation was observed in the median estimates of EPGs among the categories of sex (*p* = 0.11 ) (Table 1). We presented the statistical relationship between individual categories under each factor variable in Table S1.

**TABLE 1:** Univariate association between EPGs of gastrointestinal parasites in cattle of Chattogram and the selected factors.

			Eggs			
Factor	Category	N	Mean	Median	Min‐Max	*p* (KW)
Production type	Commercial (large scale)	49	404.1	300	100–1600	<0.01
	Commercial (medium scale)	30	140	100	50–300	
	Household (small scale)	28	285.7	200	100–1000	
Breed	Cross (Holstein × Friesian)	91	283.5	200	50–1600	0.84
	Indigenous	12	391.7	250	200–1000	
	Hariana	1	300	300	300	
	Shahiwal	3	400	100	100–1000	
Animal class	Bull	18	355.6	200	100–1300	<0.01
	Heifer	1	100	100	100	
	Calf	8	450	250	100–1100	
	Milch cow	60	218.3	200	50–1000	
	Pregnant heifer	4	500	450	200–900	
	Pregnant dry cow	4	325	350	100–500	
	Pregnant milch cow	12	458.3	300	100–1600	
Sex	Male	21	357.1	200	100–1300	0.34
	Female	83	278.3	200	50–1600	
	Missed	3	466.7	200	100–1100	
Deworming status	Yes (30–120 days)	80	318.8	200	50–1600	<0.01
	No	27	240.7	200	100–1000	

Abbreviations:KW: Kruskal–Wallis;Max, maximum; Min, minimum.

### 3.3 Frequency distribution of eggs of gastrointestinal parasites in relation to farm production and parasite types

The PP of trematode eggs was 69.1% in cattle of medium‐scale farms, 65% in cattle of small‐scale farms (household farms), and 47.9% in cattle of large‐scale farms. These findings varied significantly (*p* < 0.05). Nematode eggs were significantly higher in cattle of medium‐scale farms (32.7%) compared to cattle of small‐scale or household farms (20.0%) or large‐scale farms (2.2%) (*p* < 0.05). Cestode eggs were more prevalent in cattle of small‐scale or household farms (10.0%) than in cattle of large‐scale farms (3.2%) (*p* < 0.05), whereas protozoa eggs were more prevalent in cattle of large‐scale farms (45.7%) than in cattle of medium‐scale farms (14.5%) (*p* < 0.05). No cestode eggs were found in cattle of medium‐scale farms and no protozoa eggs were found in cattle of small‐scale or household farms.

*Paramphistomum* spp. eggs, irrespective of farming systems, were more prevalent (25.5%−56.4%) than any other parasitic eggs. *Trichostrongylus* spp. in cattle of small‐scale (household farms) and *Balantidium coli* (*B. coli*) in cattle of large‐scale farms had the highest percentage of parasitic eggs, 15.0% and 37.2%, respectively. *Ascaridia* spp. was the least prevalent parasitic egg (0%−1.1%) among all identified, irrespective of farming types (Table 2).

**TABLE 2:** Frequency distribution of parasite eggs by class and genus, and cattle farm production type in Chattogram.

Class of eggs of parasites		Commercial (large scale) No. of samples tested: 94 (*n*, %)	Commercial (medium scale) No. of samples tested: 55 (*n*, %)	Household (small scale) No. of samples tested: 40 (*n*, %)	
Nematode	Genus	2 (2.2)	18 (32.7)	8 (20.0)	*p*
	*Ascaridia* spp.	1 (1.1)	1 (1.8)	0	0.01
	*Bunostomum* spp.	1 (1.1)	0	0	
	*Haemonchus* spp.	0	6 (10.9)	2 (5.0)	
	*Ostertagia* spp.	0	2 (3.6)	0	
	*Trichuris* spp.	0	2 (3.6)	0	
	*Trichostrongylus* spp.	0	7 (12.7)	6 (15.0)	
Trematode		45 (47.9)	38 (69.1)	26 (65.0)	
	*Paramphistomum* spp.	24 (25.5)	31 (56.4)	12 (30.0)	<0.01
	*Schistosoma* spp.	21 (22.3)	7 (12.7)	14 (35.0)	
Cestode		3 (3.2)	0	4 (10.0)	
	*Moniezia* spp.	0	0	4 (10.0)	NA^a^
	*Taenia* spp.	3 (3.2)	0	0	
Protozoa		43 (45.7)	8 (14.5)	0	
	*Blantidium coli*	35 (37.2)	5 (9.1)	0	NA^a^
	*Coccidia* spp.	8 (8.5)	3 (5.5)	0	

Abbreviation: NA, not available.

^a^ Due to the presence of a “0” value in a category, the *p*‐value could not be estimated.

### 3.4 Single to multiple infection in cattle by different gastrointestinal parasites

The cattle in a medium‐scale commercial production system predominantly experienced single parasitic infections, totaling 84 cases. Conversely, double infections were more prevalent in cattle on the large‐scale commercial farms, with a total of 33 cases. Unlike other production systems, large‐scale production system cattle exhibited both triple and quadruple infections. An overall predominance of *Paramphistomum* spp. and *B. coli* was observed in both single and multiple infections, irrespective of production systems (Table 3).

**TABLE 3:** Frequency distribution of the level and types of gastrointestinal parasites in different cattle production systems in Chattogram.

Farm	Single infection	N	Double infection	*n*	Triple infection	*n*	Quadruple infection	N
Commercial (large scale)	*Paramphistomum* spp.	7	*B. coli* and *Coccidia* cyst	1	*B. coli*, *Paramphistomum* spp. and *Schistosoma* spp.	1	*Bunostomum* spp., *B. coli*, *Schistosoma* spp. and *Paramphistomum* spp.	1
	*Schistisoma* spp.	8	*B. coli* and *Paramphistomum* spp.	9	*B. coli*, *Coccidia* cyst and *Schistosoma* spp.	3	*B. coli*, *Coccidia* cyst, *Paramphistomum* spp. and *Schistosoma* spp.	2
	*Coccidia* cyst	2	*Schistosoma* spp. and *Paramphistomum* spp.	3				
	*B. coli*	14	*B. coli* and *Schistosoma* spp.	3				
	*Ascaridia* spp.	1	*B. coli* and *Taenia* spp.	1				
	*Taenia* spp.	2						
	Unknown	2						
	N	36		17		4		***3***
Commercial (medium scale)	*Paramphistomum* spp.	23	*Paramphistomum* spp. and *Coccidia* cyst	1	*Haemonchus* spp., *Schistosoma* spp. and *Paramphistomum* spp.	1		
	*Schistosoma* spp.	3	*B. coli* and *Paramphistomum* spp.	1				
	*Coccidia* cyst	2	*B. coli* and *Schistosoma* spp.	1				
	*B. coli*	2	*Trichostrongylus* spp. and *Haemonchus* spp.	2				
	*Ostertagia* spp.	2	*Paramphistomum* spp. and *Haemonchus* spp.	2				
	*Trichostrongylus* spp.	3	*Schistosoma* spp. and *Paramphistomum* spp.	3				
	*Haemochus* spp.	2						
	*Trichuris* spp.	1						
	N	38		10		1		0
Small scale (household)	*Paramphistomum* spp.	1	*Schistosoma* spp. and *Paramphistomum* spp.	2				
	*Schistosoma* spp.	3	*Paramphistomum* spp. and *Trichostrongylus* spp.	1				
	*Trichostrongylus* spp.	2	*B. coli* and *Trichostrongylus* spp.	1				
	*Haemonchus* spp.	1	*Paramphistomum* spp. and *Moniezia* spp.	1				
	*Trichuris* spp.	1						
	*Ascardia* spp.	1						
	*Moniezia* spp.	1						
	N	10		5		0		0
	Overall total	84		33		5		3

### 3.5 Univariate risk factors for gastrointestinal parasites in cattle (yes/no, based on McMaster technique) and the selected factors

The likelihood of having a GIP infection was 16.5 times (95% confidence interval [CI]: 4.7−57.5) higher in the combined category of ‘pregnant dry and milch cow’ than that of a calf (*p* = 0.001). The odds ratio was 11.2 (95% CI: 3.5−35.7) in bull and heifer having GIP infection compared to calf (p ≤ 0.001). Moreover, the odds of harbouring GIP infection were 6.7 times (95% CI: 2.8−16.0) higher in milch cows than in calves (*p* < 0.001). The odds of GIP infection were 4.6 times (CI: 0.9−21.3) more likely in indigenous breeds than in crosses (*p* = 0.05). Dewormed cattle had a higher prevalence of GIP infection than non‐dewormed cattle (OR = 2.3; CI: 1.2−4.3; *p* = 0.01) (Table 4).

**TABLE 4:** Univariate risk factor analysis between a binary outcome of gastrointestinal parasites and the selected factors.

Factor	Category	N	Status of GIPs		*p*	OR	95% CI	*p*
			Yes (%)	No.				
Farm type	Commercial (large and medium scale)	149	79 (53.0)	70		1		
	Household (small scale)	40	28 (70.0)	12	0.07	2.1	0.9–4.3	0.5
Animal class	Calf	41	8 (19.5)	33		1		
	Bull and heifer	26	19 (73.1)	7		11.2	3.5–35.7	<0.001
	Milch cow	97	60 (61.9)	37		6.7	2.8–16.0	<0.001
	Pregnant dry and milch cow	25	20 (80.0)	5	<0.001	16.5	4.7–57.5	<0.001
Breed	Cross	176	96 (54.6)	80		1		
	Indigenous	13	11 (84.6)	2	0.04	4.6	0.9–21.3	0.05
Sex	Male	31	21 (67.7)	10		1		
	Female	131	83 (63.4)	48	0.7	0.8	0.4–1.9	0.6
Deworming	No	63	27 (42.9)	36				
	Yes	126	80 (63.5)	46	0.008	2.3	1.2–4.3	0.01

Abbreviations: CI, confidence interval; GIPs, gastrointestinal parasites; OR, odds ratio.

## 4 DISCUSSION

In this study, we used three different methods for the detection and quantification of GIP's existence among the sample. The sedimentation‐floatation method is reported to have a sensitivity of approximately 72.4% (Tomczuk et al., 2014) for detecting helminth eggs or larvae, as well as protozoan parasites. However, the sensitivity may differ depending on the parasite being targeted. Additionally, the modified McMaster method enables the quantification of the parasite load by estimating the number of parasite eggs or oocysts per gram of faeces. Contrary to their specificity, parasitic organisms can be easily distinguished from trash and other non‐parasitic materials via the sedimentation‐floatation method. However, each approach calls for a skilled technician and interpreter. We employed all these methods in our study to minimize the likelihood of missing any GIP types in the sampled animal faeces.

Overall, the prevalence of GIPs was high regardless of production types, aligning with findings from numerous previous studies on cattle in similar production systems across the world (Abraham et al., 2017; Chowdhury et al., 2017; Huang et al., 2014; Keyyu et al., 2006). Earlier studies reported 37.0% GIPs in cattle on small‐scale farms (household farms), 44.4% in cattle on medium‐scale commercial farms, and 67.0% in cattle on large‐scale commercial farms. Remarkably high GIP prevalence and moderate‐to‐heavy infection load (101−16,000 EPG per individual) in small‐scale household cattle in the present study could be due to free‐range grazing on untreated pastureland, vectors, season, no or inconsistent deworming schedule, and the presence of environments that parasites like, such as low land and stagnant drainage systems, in the study sites. Cattle in medium‐ and large‐scale systems may have a high GIP prevalence but a lower infection load (at least 100 EPG per individual) than cattle in small‐scale systems. This could be because deworming is not done often enough or because worms are becoming resistant to anthelmintics. Anthelmintic resistance in GIPs is common as anthelmintics are being used indiscriminately in third‐world countries (Chinyelu, 2018; Samad et al., 2020).

Multivariate logistic regression was not conducted in this study due to multicollinearity among the variables. In the present study, a number of risk factors were determined for the presence of GIP infection in cattle. Pregnant and milch cows had significantly higher odds of GIP infection, which may be due to poor immunological status during pregnancy and milking, along with missing anthelmintic dosing (Getahun et al., 2017). Aged animals (heifers and bulls) have higher odds of GIP infection, likely to have occurred because of a wider exposure time for parasites (Bharathi & Kumar, 2017; Rashid et al., 2015). Indigenous cattle had a greater likelihood of GIP infection in contrast to cross‐bred cattle due to being reared in a traditional household rearing system with poor hygienic conditions, allowed to graze on untreated pasture land, and usually not being dewormed (Nath et al., 2016; Rathore et al., 2017). A previous study in Nepal, a neighbouring country of Bangladesh, has also indicated similar risk factors (Yadav et al., 2015). Furthermore, in Bangladesh, cross‐bred cattle are predominantly reared in commercial farming systems utilizing zero‐grazing techniques, which could potentially act as a protective factor against parasitosis (Stadalienė et al., 2014). It is surprising that dewormed cattle had significantly higher odds of GIP infection, which could be due to having developed anthelmintic resistance against GIPs. There are many reasons for anthelmintic resistance, such as indiscriminate use of anthelmintics, dosing inconsistencies, and low‐quality drugs (Ahaduzzaman, 2016; Chaudhry et al., 2015; Kumar et al., 2017). Although our study primarily focused on some particular risk factors associated with parasitosis in cattle, it is important to acknowledge that there are other potential factors that merit further investigation. For instance, the influence of selective breeding practices and nutritional status on the development of parasitosis should be examined; however, these aspects were not included in the scope of this study (Ahmad et al., 2023; Dorny et al., 2011). The exploration and analysis of additional factors could provide a more comprehensive understanding of the complexities related to parasitosis in cattle, and we intend to consider these aspects in our future research.

A wide range of parasite eggs belonging to different classes were identified in the current study, among which eggs of *Paramphistomum* spp., *Schistosoma* spp., *Trichostrongylus* spp., *Haemonchus* spp., and *Ostertagia* spp. were more commonly found in cattle of medium‐scale rearing systems. These findings are supported by many studies (Akanda et al., 2014; Alim et al., 2012; Islam et al., 2015; Shit et al., 2017). The possible reasons for the results might be inconsistency in washing fodder or grasses, the presence of parasite vectors in pasture land, or mixed farming with goats or other livestock species (Murthy & Rao, 2014).

Regardless of production type, cestode infection with *Moniezia* spp. or *Taenia* spp. was quite low in this study, which suggests unsuitable ecology (such as the presence of vectors) for cestodes in the study areas. However, eggs of *Moniezia* spp. and *Taenia* spp. were detected in cattle in other studies in Bangladesh and overseas (Akanda et al., 2014; Alim et al., 2012).

Protozoal infections (*B. coli* and *Coccidia* spp.) were quite common in cattle in commercial production systems. The potential reasons could be the location of farms (hilly areas), high stock density, and poor personal and environmental hygiene (Nath et al., 2016).

Infection with single parasites (such as *B. coli*, *Paramphistomum* spp., and *Schistosoma* spp.) was more prevalent across different production systems (Nath et al., 2016). However, concurrent infections with double and triple parasites of *B. coli*, *Paramphistomum* spp., and *Schistosoma* spp. also occurred. Similar patterns of parasitic infection in cattle were previously reported nationally and internationally (Rahman & Samad, 2010). Animals infected with a single parasite of different types share a common environment, which can facilitate concurrent infection. There is also a possibility for animals to contract multiple infections by sharing an environment infested with multiple parasites.

## 5 CONCLUSION

The prevalence of GIPs among cattle population in Chattogram is notably high with variations observed across different scales of farming. There is a higher prevalence and heavier infection load in small‐scale farms, attributed to inconsistent deworming and grazing practices. Conversely, large‐scale farms tend to have a lighter infection load. Pregnant and milch cows, as well as indigenous cattle, have higher odds of having GIPs. Anthelmintic resistance is a likely reason why dewormed cattle have higher odds of having a GIP infection. Protozoal (*B. coli* and *Coccidia* spp.), nematode, and trematode infections prevail across all farm types, with a single parasite infections being the most common. More judicious use of anthelmintics and testing for anthelmintic resistance are key to tackling the high prevalence of GIPs and preventing future infections. The development of an anthelmintic regimen that prevents both underdosing and overdosing, incorporating a combination of products, and treating only those animals in need are crucial strategies to combat anthelmintic resistance effectively.

## 6 LIMITATIONS

The limitations of the study are as follows:

1. Farms were categorized based on herd size: those with more than 24 cattle were considered large‐scale farms; those with a herd size ranging minimum of 8 to a maximum of 24 cattle were considered medium‐scale farms, and those with a herd size ranging from a minimum 2 to a maximum of 5 were considered small‐scale household farms. The inclusion of farms in the study also depended on farmers’ willingness to participate. Although the animals were selected randomly within the farm, we cannot deny the chance of selection bias.
2. A little information bias might be introduced. The expert epidemiology team was involved in data and sample collection.
3. There is no chance of diagnostic errors, but some technical problems might have happened due to the involvement of more than one assistant or researcher in lab work.
4. Our study used specific diagnostic techniques including direct smear, floatation, sedimentation, and modified McMaster techniques, which may not cover the entire spectrum of parasitic infections. In our future study, we will explore the integration of more comprehensive diagnostic methods to enhance accuracy.

## AUTHOR CONTRIBUTIONS

Md. Abu Sayeed: Data curation, formal analysis, investigation, methodology, software, validation, visualization, writing—original draft, writing—review and editing. Lauren Ungar: Data curation, formal analysis, methodology, writing—original draft, writing—review and editing. Yeasin Haider Chowdhury: Formal analysis, investigation, validation, visualization, writing—review and editing. Md. Saiful Bari: Software, validation, visualization, writing—review and editing. Md. Mizanur Rahman: Resources, supervision, validation, writing—review and editing. Sawkat Answer: *Funding acquisition, resources, supervision, validation, writing—review and editing*. Md. Ahasanul Hoque: Conceptualization, formal analysis, funding acquisition, methodology, project administration, resources, supervision, validation, visualization, writing—review and editing.

All authors have read and agreed to the published version of the manuscript.

## CONFLICT OF INTEREST STATEMENT

The authors declare no conflicts of interest.

## FUNDING INFORMATION

The authors declare that this research received no specific funding.

### ETHICS STATEMENT

We did not follow any invasive procedure for sampling to avoid any types of animal welfare and ethical concerns. We took verbal concerns from the farmers before sample collection. We have maintained a standard procedure for sample collection that described in the methodology section of the article. The study was approved by the CVASU Ethics Committee, Bangladesh (permit reference number: EC/2018/39[2/9]; date: 15 May 2018).

### PEER REVIEW

The peer review history for this article is available at https://www.webofscience.com/api/gateway/wos/peer-review/10.1002/vms3.1325.

## Supporting information

Supporting Information

## Data Availability

All the data that was generated during this study has been presented in the result section of the study.
