# Supplementary material for: Gastrointestinal parasitosis in cattle: Unveiling the landscape across diverse production systems in Bangladesh
Source: Vet Med Sci. 2023 Nov 27;10(1):e1325. doi: 10.1002/vms3.1325 (PMC10766017; doi:10.1002/vms3.1325)
Supplement: Supplementary file 1 — Supporting Information [file VMS3-10-e1325-s001.docx]

**Suppl Table 1:** Post-hoc test among the selected factors used in univariate association between the status of EPGs of gastrointestinal parasites per gram faeces in cattle of Chattogram.

|  | Commercial (Large-scale) *(p-value)* | Commercial (Medium scale) *(p-value)* |  | | | |
| --- | --- | --- | --- | --- | --- | --- |
| Commercial (Medium scale) | <0.01 |  |  | | | |
| Household (Small-scale) | 0.02 | 0.003 |  | | | |
|  | Cross (Holstein × Friesian) *(p-value)* | Indigenous *(p-value)* | Hariana *(p-value)* |  | | |
| Indigenous | 0.49 |  |  |  | | |
| Hariana | 0.24 | 0.26 |  |  | | |
| Shahiwal | 0.29 | 0.31 | 0.19 |  | | |
|  | Bull *(p-value)* | Heifer *(p-value)* | Calf *(p-value)* | Milch cow *(p-value)* | Pregnant heifer *(p-value)* | Pregnant dry cow *(p-value)* |
| Heifer | 0.05 |  |  |  |  |  |
| Calf | 0.02 | 0.22 |  |  |  |  |
| Milch cow | <0.01 | 0.17 | 0.35 |  |  |  |
| Pregnant heifer | 0.17 | 0.02 | 0.01 | <0.01 |  |  |
| Pregnant dry cow | 0.49 | 0.06 | 0.08 | 0.08 | 0.23 |  |
| Pregnant milch cow | 0.29 | 0.03 | 0.01 | <0.01 | 0.29 | 0.37 |
|  | Male *(p-value)* | Female *(p-value)* |  | | | |
| Female | 0.07 |  |  | | | |
| Missed | 0.36 | 0.4021 |  | | | |
